# A shape-anisotropic reflective polarizer in a stomatopod crustacean

**DOI:** 10.1038/srep21744

**Published:** 2016-02-17

**Authors:** Thomas M. Jordan, David Wilby, Tsyr-Huei Chiou, Kathryn D. Feller, Roy L. Caldwell, Thomas W. Cronin, Nicholas W. Roberts

**Affiliations:** 1 School of Biological Sciences, University of Bristol, Tyndall Avenue, Bristol, BS8 1TQ, UK; 2 Bristol Centre for Functional Nanomaterials, School of Physics, HH Wills Physics Laboratory, University of Bristol, Tyndall Avenue, BS8 1TL, UK; 3 Department of Life Sciences, National Cheng Kung University, Tainan City 70101, Taiwan, Republic of China; 4 Department of Integrative Biology, University of California, Berkeley, CA 94720, USA; 5 Department of Biological Sciences, University of Maryland Baltimore County, 1000 Hilltop Circle, Baltimore, Maryland 21250, USA

## Abstract

Many biophotonic structures have their spectral properties of reflection ‘tuned’ using the (zeroth-order) Bragg criteria for phase constructive interference. This is associated with a periodicity, or distribution of periodicities, parallel to the direction of illumination. The polarization properties of these reflections are, however, typically constrained by the dimensional symmetry and intrinsic dielectric properties of the biological materials. Here we report a linearly polarizing reflector in a stomatopod crustacean that consists of 6–8 layers of hollow, ovoid vesicles with principal axes of ~550 nm, ~250 nm and ~150 nm. The reflection of unpolarized normally incident light is blue/green in colour with maximum reflectance wavelength of 520 nm and a degree of polarization greater than 0.6 over most of the visible spectrum. We demonstrate that the polarizing reflection can be explained by a resonant coupling with the first-order, in-plane, Bragg harmonics. These harmonics are associated with a distribution of periodicities perpendicular to the direction of illumination, and, due to the shape-anisotropy of the vesicles, are different for each linear polarization mode. This control and tuning of the polarization of the reflection using shape-anisotropic hollow scatterers is unlike any optical structure previously described and could provide a new design pathway for polarization-tunability in man-made photonic devices.

There is a great diversity of reflective photonic structures throughout the animal kingdom, including biological analogues of periodic photonic crystals^1^,^2^,^3^,^4^, quasi-ordered amorphous solids^5^,^6^,^7^,^8^, and one-dimensional multilayer reflectors^9^,^10^,^11^,^12^. Biophotonic structures frequently serve as optical adaptations that enable animals to communicate through reflective visual signals. It is therefore highly advantageous to be able to control the optical properties of these reflectors and thus the visual information content of the reflection. For example, multilayer reflectors found in fish and cephalopods are ‘spectrally tuned’ via the distribution of layer thicknesses around the quarter-wave criteria to have colours that range from blue to silver^9^,^12^,^13^. However, whilst the principles that control and optimize either the reflected colour or the level of reflectivity are understood, the same cannot be said for how 3-dimensional animal photonic structures could be structured to control the polarization of light.

At normal incidence, the polarization of the light reflected from the majority of photonic structures, whether biological or man-made, remains unchanged. In order to polarize the reflection, the symmetry of the photonic structure with respect to orthogonal polarization modes must be broken. For example, the circularly polarized reflections seen from the chitin structures in the elytra of beetles^14^ and cholesteric or chiral smectic liquid crystals^15^ arise due to chirality. In two-dimensional photonic crystals, it is a general result that for light propagation along correctly chosen coordinate axes it is possible to separate orthogonal polarization modes^16^,^17^ and geometric shape-anisotropy can be introduced to provide polarization-selective reflection and transmission^17^. In true 3-dimensional photonic crystals, however, it is in general not possible to obtain the strict separation of orthogonal polarization modes^16^. Subsequently, engineering a three-dimensional photonic structure to produce a reflection with a high degree of polarization mode separation is a non-trivial task.

One 3-dimensional biophotonic structural reflector that potentially acts as a linear polarizer is found in the maxilliped appendages of certain species of stomatopod crustaceans (known commonly as mantis shrimps). Maxillipeds are frontal sets of modified limbs, generally used to manipulate food or for cleaning, and are involved in sexual and agonistic communication^18^,^19^,^20^. In the genus *Haptosquilla*, the first maxillipeds possess a striking and conspicuous blue/green colouration, which in some species is also strongly linearly polarized^21^,^22^. Figure 1a,b illustrates two species that display both blue/green and polarized reflections of the first maxillipeds, *H. trispinosa* (a) and *H. banggai* (b).

Previous work^21^,^22^ hypothesised that the blue/green polarizing reflections arise from a quasi-ordered structure found under the cuticular surface of the maxillipeds. The three-dimensional architecture comprises a morphology of ovoid vesicles that exhibit degrees of both positional and orientational order^21^,^22^. In this paper, we use a combination of transmission electron microscopy, optical measurements, and theoretical modelling to validate that the reflection arises from this structure, which we categorise as a ‘shape-anisotropic amorphous solid’. Our theoretical model, which is based upon decomposing the optical response of the structure into contributions from different Bragg harmonics, demonstrates that the polarizing reflection can be explained by a resonant coupling between incident light and the in-plane (first-order) Bragg harmonics^23^,^24^. The first-order Bragg harmonics arise due to the in-plane periodicity from the spacing between the interior walls of the hollow ovoid vesicles, and, due to the in-plane shape-anisotropy, are different for each linear polarization mode. This in-plane coupling to a first-order Bragg harmonic has not been reported as a mechanism of reflection before in a biophotonic structure. To the best of our knowledge, the apparent ‘tunability’ of the polarization properties of reflection via the in-plane dimensions of the hollow vesicles provides a novel design pathway to control the polarization properties of reflection in a 3-dimensional photonic structure.

## Materials and Methods

### Animals

*H. trispinosa* were collected from the intertidal region at Lizard Island Research Station (Queensland, Australia) under collection permit (Queensland–GBRMPA permit G12/35042.1).

### Reflectance spectrophotometry

**Figure 2:**
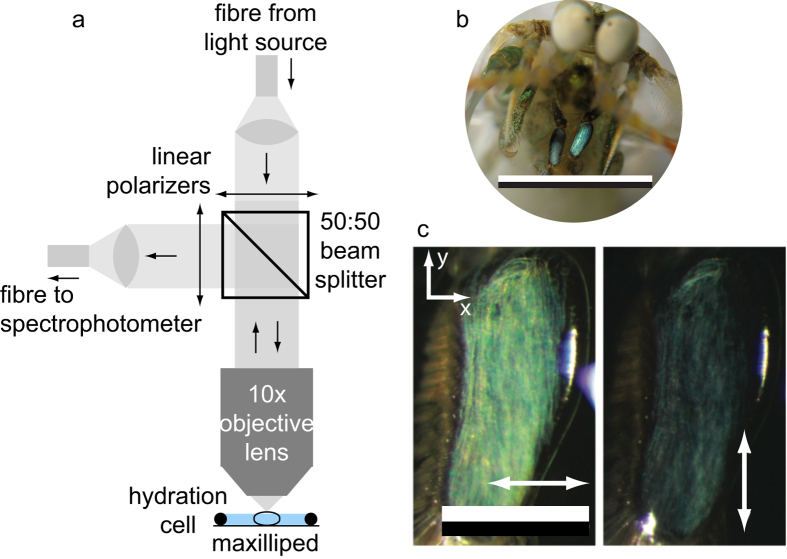
Experimental setup to measure the polarized reflectivity of the first maxillipeds of *Haptosquilla trispinosa.* (**a**) Schematic diagram of the experimental setup used to measure the spectral reflections from the maxillipeds as a function of input and output polarization. (**b**) Optical micrograph of an individual *H. trispinosa* and the maxillipeds. Scale bar 10 mm. (**c**) Close up of the maxillipeds with an analyser placed horizontally (left) and vertically (right). Scale bar 1 mm.

Specimens were euthanized in an ice slurry and maxillipeds removed under a dissection microscope. Reflectance spectra were measured using an optical system that included a white LED light source (Ocean Optics, FL, USA), a glass polarizer and analyser with transmission axes aligned parallel or perpendicular to the major-axis of the maxilliped (Edmund Optics, NJ, USA), 50:50 non-polarizing beam-splitter (ThorLabs, NJ, USA), 10 × magnification microscope objective (NA = 0.25, Olympus, Tokyo, Japan) and spectrophotometer (Ocean Optics, FL, USA) (Fig. 2a,b). Reflection spectra were measured from a circular area on the maxillipeds (Fig. 2c) of approximately 10 μm in diameter using 4 different polarizer and analyser orthogonal polarization mode combinations. Samples were kept for the duration of measurement in seawater in a hydration cell comprised of a microscope cover slip and rubber o-ring sealed with vacuum grease.

### Electron Microscopy sample preparation and measurements

Dissected maxillipeds were fixed with 2.5% glutaraldehyde in PEMS buffer (0.1 M PIPES, 0.01 M EGTA, 0.0005 M MgCl_2_, 0.15 g/ml sucrose, pH = 7.1). The samples were fixed overnight at 4 °C and post fixed with 1% osmium tetroxide in PEMS for 2 hours on ice. Specimens were stained *en bloc* with 2% uranyl acetate in absolute ethanol for 2 hours at room temperature during dehydration. Finally a gradient of 1:1 (w/w) Epon:Spurr’s resins in acetone at room temperature were used to infiltrate the samples. These were cured under a slight vacuum in 100% of the same resin for 24 hours at 70 °C. Sections of 60~90 nm thickness were prepared from the specimen blocks. These thin sections were examined on a Zeiss transmission electron microscope (EM-10CA) with an accelerating voltage of 60~80 kV.

### Image analysis and prediction of reflection spectra

The development of the methodology and theoretical basis for calculating the reflection spectra of the stomatopod polarizer follows our classification of the structure as a shape-anisotropic amorphous solid, and is worked through in detail in the results and discussion section. The method that we develop is an integration of the works of Prum and colleagues^5^,^6^,^25^ on the optics of quasi-ordered biophotonic reflectors, and the theory of photonic crystal slabs^23^,^24^. The method relates the relative frequency distributions for the sets of in-plane periodicities to the polarized reflection spectra, and the digital image analysis procedure required for replication of this method is described below.

For the analysis, transmission electron micrograph sections from the sagittal plane were imported into Image J. Curves, which were assumed to represent the centre of the scattering interface, were then traced along the centre of the ovoid vesicle walls using the freehand analysis tool. The geometric data were stored as a binary mask in which the vesicles were represented as solid objects. This first binary mask was then imported into MATLAB and differentiated to produce a second binary mask in which the vesicles were represented as hollow objects (with the two dimensional edges represented by 1s and the interior and exterior of the vesicles represented by 0 s). In this representation the ovoid vesicles are treated as ‘extended’ scattering objects, with each black pixel acting as a scattering interface. The relative frequency distributions along each Cartesian coordinate axis were obtained by line-scanning the second binary mask and measuring the sets of distances between black pixels. A 10 nm bin width was used when binning the relative frequency distributions with the interval [0,1000] nm used for normalisation. Two separate TEM sections were used which provided a total sample of ~350 vesicles.

## Results and Discussion

### Optical measurements

Unlike previous measurements of reflection spectra from the first maxillipeds^22^, reflection spectra for four orthogonal polarization mode combinations: *R*_*xx*_, *R*_*yy*_, *R*_*xy*_ and *R*_*yx*_ were made from 400–700 nm under normally incident illumination. The *x*-axis (depicted in Fig. 2c) corresponds to the horizontal direction across the short axis of the maxillipeds. The *y*-axis is the vertical direction and parallel to the long axis of the maxillipeds and the *z*-axis is normal to the surface of the maxilliped.

**Figure 3:**
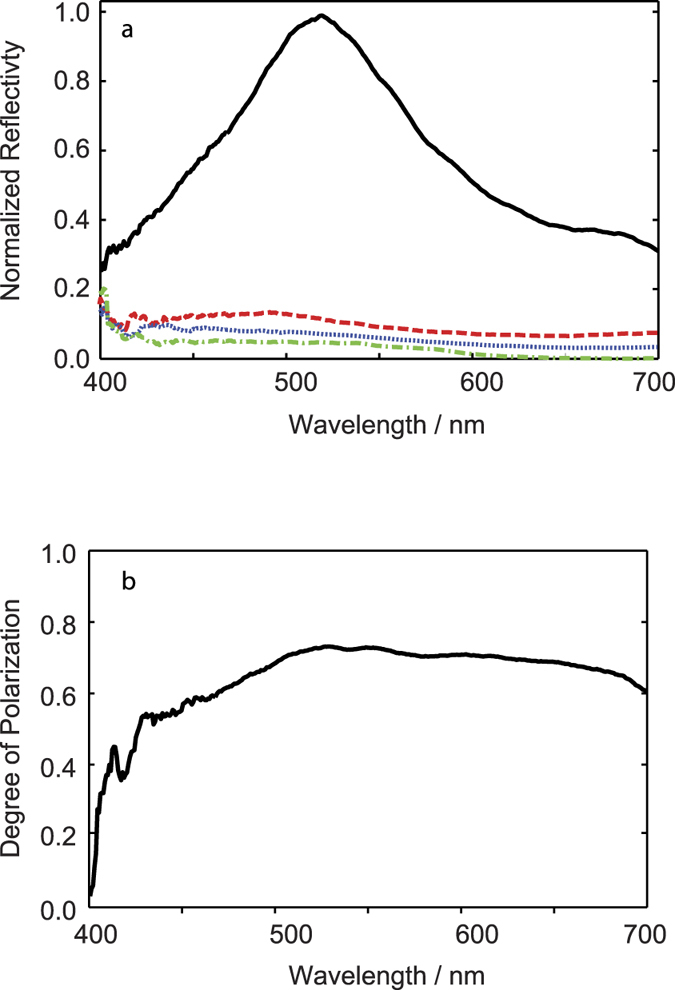
Reflection and degree of polarization spectra. (**a**) Reflection spectra for the four polarization mode combinations: *R*_*xx*_ (solid black line), *R*_*yy*_ (blue dotted line), *R*_*xy*_ (red long-dashed line), *R*_*yx*_ (green dash-dotted line). (**b**) Degree of polarization spectra for reflected light, *d*  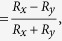 where 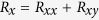 and 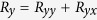.

The horizontally polarized reflection is primarily composed of the uncoupled *R*_*xx*_ polarization mode combination (Fig. 3a), which has a spectral maximum in the green region of the spectrum at ∼520 nm and a relatively broad extent across the blue/green range with the upper and lower FWHM limits at 600 nm and 450 nm respectively. The relative contributions to the overall reflectivity from the uncoupled *R*_*yy*_ polarization mode combination and the cross polarization mode combinations *R*_*xy*_ and *R*_*yx*_ are all minor compared to the *R*_*xx*_ term (Fig. 3a). Thus the maxillipeds act as efficient linear polarizers for unpolarized incident light over much of the (human) visible spectrum (Fig. 3b). The degree of polarization for reflected light, 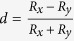, is greater than 0.6 over the wavelength range from 470 nm to 700 nm. It should be noted that there is no ellipticity in the reflected light as measured in previous studies^21^,^22^.

### Morphology and classification of the photonic structure

**Figure 4:**
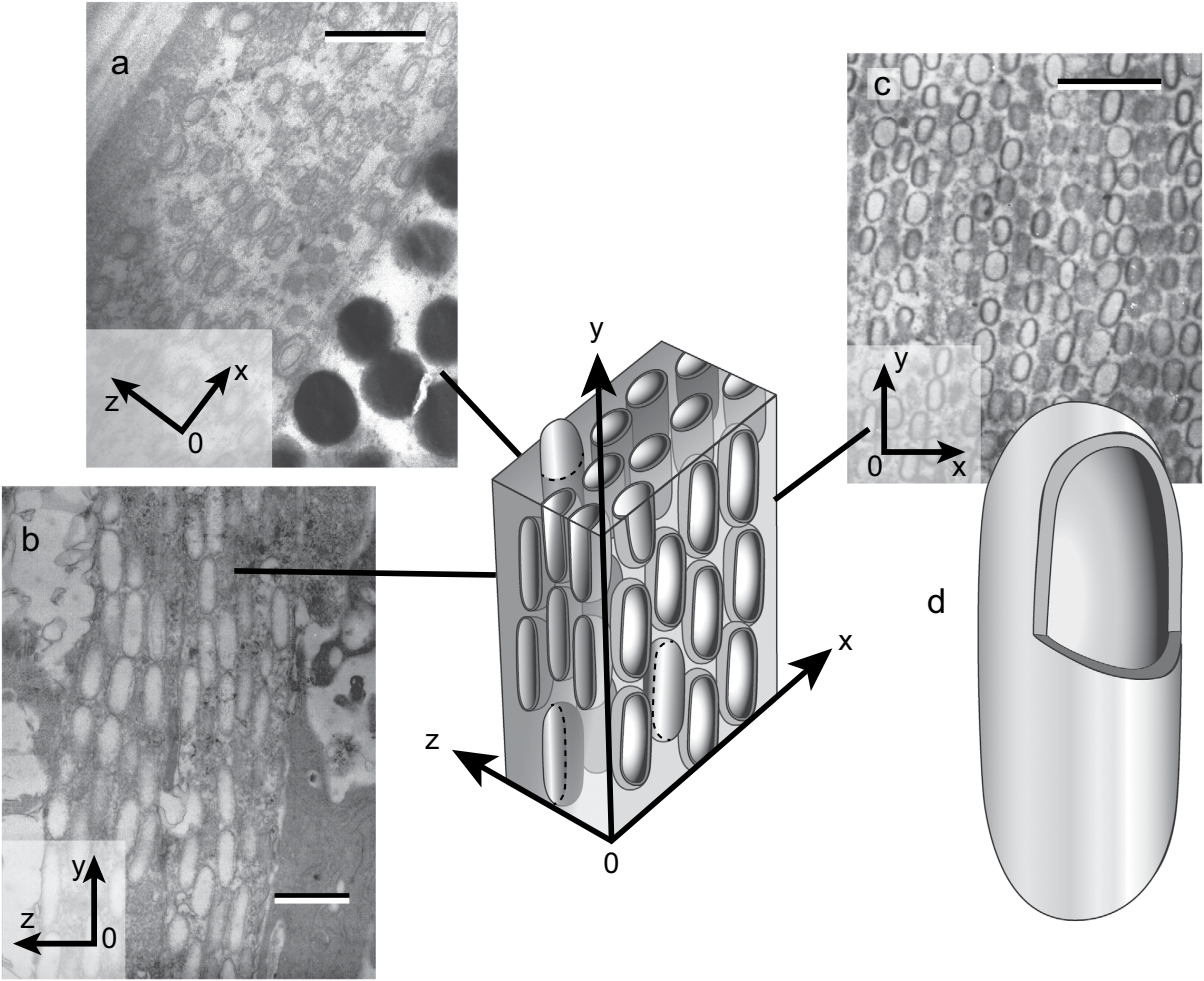
The morphological structure of the ovoid vesicles in the maxilliped. Transmission electron micrographs of: (**a**) a cross section through the structure (the *x-z* plane), scale bar 0.5 μm, (**b**) the sagittal plane of the maxilliped (the *y-z* plane), scale bar 0.5 μm, and (**c**) parallel to the front surface of the maxilliped (the *x-y* plane), scale bar 1.35 μm. (**d**) An illustration depicting the 3-dimensional relationship of the TEMs (**a**–**c**) and the orientation, shape-anisotropy, and hollow structure of the vesicles.

A series of detailed transmission electron micrographs (TEMs) (Fig. 4a–c) were made from individual maxillipeds, taking sections of the three main principal orthogonal planes: a cross-sectional plane (Fig. 4a), a sagittal plane through the maxilliped (Fig. 4b), and a plane parallel to the maxilliped surface (Fig. 4c). A coordinate system convention has been applied as follows (and matches the axes in Fig. 2c): the *x-y* plane is aligned locally parallel to the maxilliped surface, with the *z*-axis perpendicular (the propagation direction for normally incident light) (Fig. 4d). The TEMs reveal that under the overlying transparent cuticle, there exists a quasi-ordered lattice of approximately 6 to 8 layers of ovoid vesicles (Fig. 4a–c). The overall thickness of the vesicle layer is 1 to 2 μm. The vesicles are highly shape-anisotropic with short in-plane vesicle axes (aligned with the *x*-axis of the coordinate system) of approximately 250–300 nm and long in-plane vesicle axes (aligned with the *y*-axis of the coordinate system) of approximately 550–600 nm (Fig. 4d). The vesicle axes aligned with the z-axis of the coordinate system are shorter than both the in-plane axes and are approximately 120–150 nm.

The ovoid vesicle lattice lacks crystalline order and it therefore cannot be classified as a periodic photonic crystal. Rather, as the size distribution of the ovoid vesicles appears to be unimodal, this suggests that the structure can be classified as an amorphous solid^7^,^8^,^25^. Amorphous photonic solids are quasi-ordered structures that possess ‘short-range order’ between scatterers^26^,^27^. The ovoid vesicle lattice is, however, distinct from previously described amorphous biophotonic solids^5^,^6^,^7^,^8^,^25^, in three important respects. Firstly, the vesicle scatterers are shape-anisotropic (rather than being isotropic); secondly, the long axes of the vesicle scatterers are of the order of optical wavelengths (∼500–600 nm rather than the typical ∼150–200 nm); and thirdly the TEM micrograph staining implies that the ovoid vesicles are have a shell structure with the interior of similar refractive index to the surrounding media.

### Development of the coherent Bragg scattering model

Whilst several precise theoretical methods are commonly used to analyse the optical properties of animal biophotonic structures; for example band-gap theory for photonic crystals^3^,^4^,^16^, and transfer matrix methods for multilayer reflectors^10^,^11^,^12^,^13^,^28^,^29^, most structural coloration, including the reflections from amorphous solids^5^,^6^,^25^, can be understood conceptually through the Bragg condition for phase-constructive interference^28^,^29^. A common form of the Bragg condition (which from herein we refer to as the zeroth-order Bragg harmonic) is given by


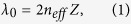


where 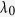 is the peak reflectivity (or ‘excitation’ wavelength), 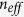
is the effective (volume averaged) refractive index and 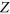 is the distance between successive scattering planes parallel to the propagation direction^5^,^6^,^25^. The zeroth-order Bragg equation reduces higher dimensional structures to a single dimension, and for reflection at normal incidence the predicted reflectivity is independent of polarization. It therefore cannot serve as an explanation for the polarizing optical response of the ovoid vesicle lattice.

Photonic structures that have their periodicity perpendicular to the propagation direction, such as photonic crystal slabs, can also generate a resonant reflection^17^,^23^,^24^,^30^,^31^. In this situation, the incident light couples to higher order Bragg harmonics that are a consequence of periodicity perpendicular to the propagation direction^23^,^24^. This resonant-coupling between the background frequency of the incident light and the frequency of the in-plane lattice periodicity is an example of a universal wave phenomenon known as a Fano resonance^32^. For normal incidence reflection, the condition for resonant-coupling between incident light and the Bragg harmonics in the plane perpendicular to the propagation direction (which from herein we refer to as the first-order Bragg harmonics) are given by


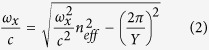


and


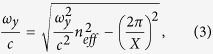


where 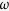 is the excitation frequency, 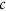 is the speed of light, 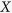 and 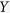 are the distances between two scattering centres perpendicular to the propagation direction along *x* and *y* axes, and the subscripts *x* and *y* indicate horizontal and vertical polarizations respectively (both as described above and denoted in Figs. 2 and 4)^24^. It is worth noting explicitly that the horizontal polarization component (aligned with the *x*-axis) interacts with y periodicity and the vertical polarization component (aligned with the *y*-axis) interacts with the *x* periodicity^33^. As, in equations (2) and (3), incident light can couple to different lattice periodicities for different incident linear polarizations, we hypothesised that this resonance process could provide an explanation for the polarizing optical response of the ovoid vesicle lattice. Using the dispersion relations for incident light, 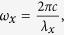 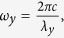 and then re-arranging equations (2) and (3), gives the following scaling relationships between the reflection/excitation wavelengths and the in-plane lattice periodicities


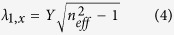


and


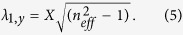


The left-subscript, 1, in equations (4) and (5) is introduced to distinguish the first-order excitation wavelengths 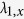 and 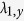 from the zeroth-order excitation wavelength 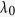.

### Analysis of reflection and polarization spectra

For amorphous biophotonic solids that are isotropic, the zeroth-order Bragg law, equation (1), has been used to theoretically predict reflection spectra that closely match experimental reflectance spectra^5^,^6^,^25^. Prum and colleagues have developed a procedure based on performing a discretised spatial frequency/periodicity decomposition of transmission electron micrograph sections. A normalised histogram representation of the reflection spectrum (expressed in units of percentage Fourier power) is then produced by using equation (1), to match the relative frequency distribution of periodicities parallel to the propagation direction, {*Z*}, to the corresponding distribution of excitation wavelengths. Whilst approximate, being based upon an effective refractive index and neglecting the effects of multiple scattering, this technique enables experimentally measured reflectance spectra to be related back to the distribution of periodicities in the photonic structure. It also serves as a demonstration that a coherent Bragg scattering process, rather than incoherent scattering processes such Tyndnall or Rayleigh scattering, is responsible for the reflection^5^,^25^.

**Figure 5:**
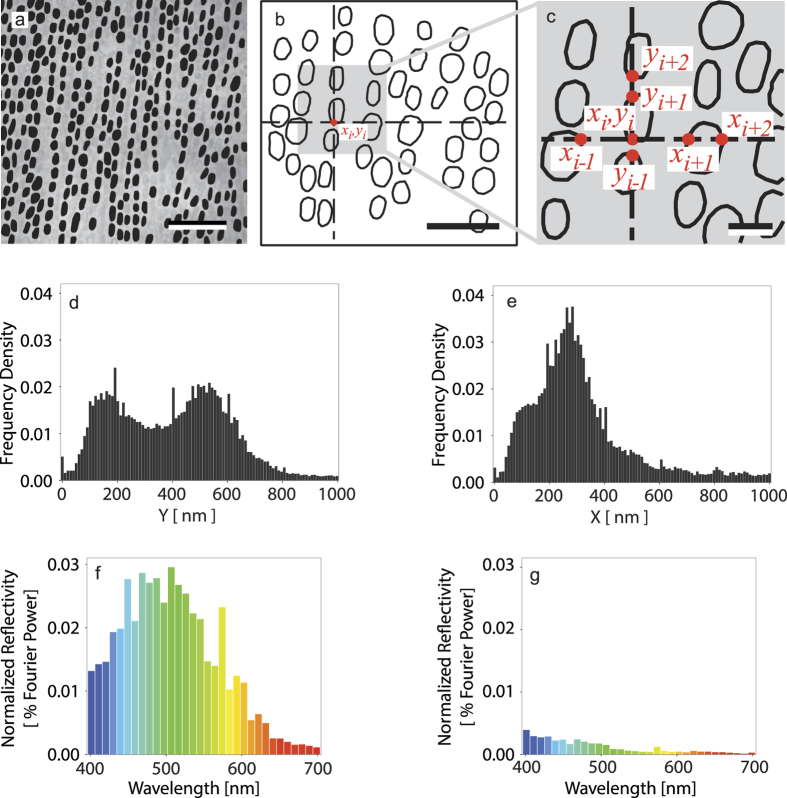
Periodicity decomposition of the ovoid vesicle lattice and predicted reflection spectra. (**a**) Thresholded image of vesicles in the plane of the maxillipeds, the *x-y* plane. Scale bar: 2.7 μm. (**b**) A differentiated binary image that represents the vesicle walls as a scattering interface. Scale bar: 2.7 μm **(c)** Expanded area of (**b**) illustrating how *x*_*i*_ and y_*j*_ define the measured sets of inter-scatterer distances, {*X*} and {*Y*}. Scale bar 2.7 μm. (**d**) Relative frequency distribution {*Y*}. (**e**) Relative frequency distribution {*X*}. (**f**) Relative reflectivity for the horizontal polarization, *R*_*x*_. (**g)** Relative reflectivity for the vertical polarization, *R*_*y*_.

In our analysis of the polarized reflection from the maxilliped structure, we extended this spatial frequency/periodicity decomposition method to include the contribution from the first-order Bragg harmonics, equations (4) and (5) This required us to calculate the relative frequency distributions for the in-plane periodicities, {*X*} and {*Y*}, (see Methods). Two different periodicity distributions are seen: {*Y*} has two peaks over the intervals ~100–200 nm and ~470–570 nm (Fig. 5d), which correspond to the sub-distributions of external and internal distances between the ovoid vesicle walls respectively. {*X*} has a broader peak over the interval ~100–350 nm, which corresponds to the combined distribution of external and internal distances between the ovoid vesicle walls (Fig. 5e).

The relative reflection spectra for the horizontal and vertical polarization modes, Fig. 5f,g, were produced by taking the square of the distributions in Fig. 5d,e respectively, and rescaling by the factor 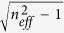 with 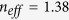, as per equations (4) and (5). This procedure is as described by Prum and Torres^25^, but for the first-order Bragg relationships rather than zeroth-order. The units of relative reflectivity, (referred to as percentage Fourier power in Prum and Torres^25^ and adopted here), are defined by setting the integral of the reflection spectra to equal 1. The effective refractive index value, *n*_*eff*_ = 1.38, represents the volume averaged refractive index of the vesicular material (refractive index unknown) and the background media, which here we assume to be cytoplasm (refractive index range 1.33–1.38). It is evident from the staining of the TEM images in Fig. 4, that the material volume fraction is dominated by the background media, hence our choice of value is within the range of values for cytoplasm.

Both the calculated spectral profiles for *R*_*x*_ and *R*_*y*_ reproduce the shape of the equivalent experimental measurements presented in Fig. 3a. The calculations match the wavelength of maximum reflection and the drop-off above ∼600 nm matches the observed blue/green colour of the reflection. The negligible predicted *R*_*y*_ reflection agrees with the minor optical response measured experimentally at visible wavelengths, however, there is also a sub-visible predicted reflection peak for *R*_*y*_ centered on 180 nm (not shown) that corresponds the peak in {*X*} in Fig. 5d. The periodicity parallel to the propagation direction (*z* axis) is predicted to give rise to a non-polarizing reflection due to the zeroth-order Bragg harmonic, equation (1). Following an analogous procedure as previously described for obtaining {*Y*} and {*X*}, we obtained a *z*-axis relative frequency distribution, {*Z*}, (not shown) which has a single peak at ~120 nm. From equation (1), this corresponds to a peak spectral response in the UV region of the spectrum at ∼ 250 nm, which is below the wavelength range of the experimental measurements in Fig. 3a, and in relation to the ecology of the animals, below the wavelength sensitivity of the UV photoreceptors.

## Conclusions

Numerous photonic structures, both man-made and biological, are known to reflect light because of the periodicities that exist within their architecture. For a wide class of photonic structures, those that have their optical response governed by the zeroth-order Bragg condition, control over the polarization properties of reflection is impossible at normal incidence. Here, we have demonstrated that an amorphous biophotonic structure produces a polarized reflection by the coupling of incident light to first-order, in-plane, Bragg harmonics. The control over the optical properties of reflection arises due to the distribution of in-plane periodicities, and, due to in-plane geometric anisotropy, enables the production of a highly polarized reflection. The high degree of linear polarization-mode separation exhibited by the biological polarizer is atypical of periodic 3-dimensional photonic structures, which in general do not enable the strict separation of polarization modes^16^,^31^; however also see work by Wang and Minghao^34^. A future consideration is that the methods employed in this study, principally extend those developed by Prum and colleagues^5^,^25^. However, there are other formalisms, such as an analysis of the photon density of states^8^ or the structure’s Mueller-matrix, that may prove useful in future investigations. A further thought is how the definition of the degree of polarization needs addressing carefully in different frames of reference from those used in this study^35^.

In the context of developing future bioinspired man-made photonic structures, several key ‘design features’ of this architecture are significant. Firstly, to the best our knowledge, the hollow nature of the vesicles provides a novel design pathway for maintaining the short-range order that is required for an optical response in an amorphous solid. Secondly, the in-plane shape anisotropy is the mechanism of polarization separation. Thirdly, the distribution of the in-plane periodicities relates to the broad reflection peak seen experimentally and is indicative of the peak broadening that can occur due to disorder in amorphous solids^5^,^6^,^7^,^8^,^25^,^26^.

We envisage that optical control using these design features could be tested in the future by building a bioinspired replica structure where the dielectric properties and dimensions of the scattering elements can be well defined and controlled. A further prediction of the proposed mechanism of maintaining short-range order is that (all other things being equal) shape-isotropic hollow scatterers of radius identical to the long axes of the vesicles would produce a non-polarizing reflection. Moreover, due to the apparent lack of a direct analogy to the biological structure in man-made optics, ‘amorphous photonic structures comprised of hollow scatterers’ provide a general class of photonic structure that requires further theoretical and experimental investigation.

## Additional Information

**How to cite this article**: Jordan, T. M. *et al.* A shape-anisotropic reflective polarizer in a stomatopod crustacean. *Sci. Rep.* **6**, 21744; doi: 10.1038/srep21744 (2016).

## Figures and Tables

**Figure 1:**
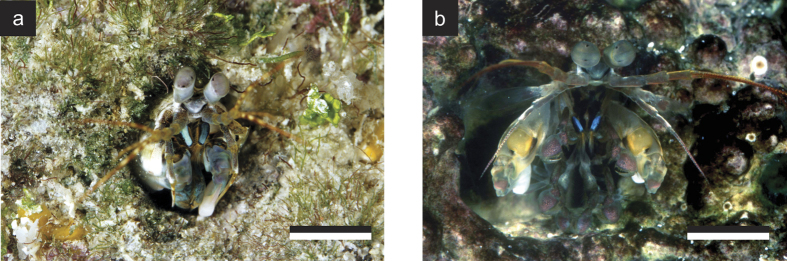
The striking polarized blue/green structural colour of the first maxillipeds in species of the stomatopod genus *Haptosquilla* (**a**) *Haptosquilla trispinosa.* Scale bar approx. 15 mm. (**b**) *H. banggai*. Scale bar approx. 10 mm.
